# A Case of Cecal Bascule Following Clostridioides difficile Colitis

**DOI:** 10.7759/cureus.93041

**Published:** 2025-09-23

**Authors:** Shea E Fincher, George Mitchell, Derek K Paul

**Affiliations:** 1 Simulation and Education Technology, Edward Via College of Osteopathic Medicine, Spartanburg, USA; 2 Critical Care Medicine, Cleveland Clinic Indian River Hospital, Vero Beach, USA; 3 Surgery, Cleveland Clinic Indian River Hospital, Vero Beach, USA

**Keywords:** bascule, cecal bascule, cecal volvulus, clostridioides difficile, large bowel obstruction

## Abstract

Cecal bascule is a rare subtype of cecal volvulus, involving the cecum rotating anteriorly onto the ascending colon. This type of cecal volvulus has no torsion component, allowing it to present uniquely compared to other bowel obstructions. Infections, inflammation, and other colonic conditions can contribute to changes in colonic motility, which may play a role in the development of cecal bascule. This is a case of cecal bascule following a colonic infection by *Clostridioides difficile*. A 62-year-old Caucasian man, with a past medical history significant for hypertension, nicotine dependence, and alcohol dependence, presented for shortness of breath. After six days in the ICU for treatment of septic shock secondary to multilobar right-sided pneumonia and new-onset atrial fibrillation with rapid ventricular response, the patient was noted to have diarrhea with positive *C. difficile* polymerase chain reaction (PCR), but with negative enzyme immunoassay (EIA) toxin. After treatment and resolution of *C. difficile* infection (CDI), an abdominal computed tomography (CT) scan noted new-onset gaseous distension of the cecum. A follow-up abdominal CT scan reported worsening cecal distension despite conservative treatment, so the patient underwent an exploratory laparotomy, which revealed a prominent cecal bascule. Postoperatively, the patient improved following cecal bascule reduction and ileocolic anastomosis, and remained clinically stable with a functional ileostomy. While CDI is commonly associated with colonic complications such as toxic megacolon and bowel obstruction, its potential role in cecal bascule remains less well understood. Cecal bascule can present uniquely with ominous symptoms, as is seen increasingly in patients with recent colonic disease. Clinicians should keep cecal bascules in their differential diagnoses when patients present with intermittent abdominal pain, as early recognition of cecal bascules is essential for timely intervention before colonic ischemia and further irreversible tissue damage occur.

## Introduction

The cecal bascule is the rarest subtype of cecal volvulus, where the distended cecum folds on a horizontal axis, anteriorly towards the ascending colon. Unlike other types of cecal volvulus, such as axial torsion and loop subtypes, cecal bascules do not include any torsion [1]. Cecal bascules account for approximately 5%-20% of all cecal volvulus cases, which in turn account for 2% of all large bowel obstructions. This condition commonly affects middle-aged adults, with a slight male predominance and an average age of 55.1 years [1].

Despite the well-known occurrence of this phenomenon since its discovery by Rokitansky in 1837, the etiopathogenesis of cecal bascules is still uncertain [1]. It is suggested that abnormal cecal motility matters in the development of cecal volvulus. Increased motility can arise from congenital causes, including failure of right colonic dorsal mesenteric fusion with the lateral parietal peritoneum during embryogenesis, or from the presence of a congenital Jackson veil. Acquired causes include exaggerated cecal motility seen after abdominal surgeries or rapid weight loss after gastric bypass surgery [2]. It is postulated that intestinal obstruction occurs because of flap valve obstruction of cecal output into the ascending colon after cecal displacement [1]. A competent ileocecal valve facilitates this obstruction by creating a closed loop between the ileocecal valve and the flexion point of the defect [1], preventing proximal decompression of the cecum [2]. Therefore, large bowel obstruction can occur in a cecal bascule without a torsional component [1,2]. The flexion of the dilated cecum compresses the anterior wall of the ascending colon, leading to possible ischemia and necrosis of the right hemicolon.

The initial presentation of a cecal bascule is highly variable because of the lack of torsional mesenteric obstruction and periodic flipping of the cecum back into its original position. Presentation can, therefore, range from classic bowel obstruction symptoms - abdominal pain, nausea, vomiting, and constipation - to intermittent abdominal cramping from periodic obstruction [3]. Entrapment of the cecum after cecal bascule formation is suggested as the final step before decompensation [2]. Therefore, it is imperative to consider cecal bascules as a differential diagnosis for presentations with abdominal pain and distension, to prevent long-term sequelae, including ischemia and necrosis of the right hemicolon and perforation of the dilated cecum [1].

Treatment of a cecal bascule is similar to that for other types of cecal volvulus, including urgent surgical intervention. Most surgically treated cecal bascules undergo a right hemicolectomy with primary ileocolic anastomosis, with or without an end-ileostomy [2].

The purpose of this report is to describe a case of cecal bascule that developed during inpatient hospitalization, after the resolution of suspected *Clostridioides difficile *colitis. Multiple case reports suggest altered colonic motility in conditions such as diverticulitis [4], inflammatory bowel disease [5], *Mycobacterium tuberculosis* [6], and other inflammatory colonic disturbances, such as cytomegalovirus colitis, can trigger the development of large bowel rotation and obstruction [7]. A case report describing the development of a cecal bascule acutely following a diagnostic colonoscopy also noted inflammation of the cecum and mucosa upon biopsy of the colon, further suggesting that colonic inflammation may be a possible contributor to the formation of cecal bascules [8]. However, cecal bascule presenting in patients with inflammatory or infectious colonic processes is uncommonly seen and reported in current literature [7].

## Case presentation

A 62-year-old Caucasian male with a past medical history significant for hypertension, nicotine dependence, and alcohol dependence presented for shortness of breath and a recent episode of upper respiratory infection lasting about a week. Chest X-ray on admission disclosed multilobar right-sided pneumonia. The patient’s laboratory values were initially notable for leukocytosis, and he was started on intravenous (IV) antibiotics and subsequently admitted to the hospital. On the first night of admission, the patient clinically deteriorated with new-onset atrial fibrillation with rapid ventricular response, extreme encephalopathy, and respiratory distress. He was transferred to the intensive care unit (ICU), where he was intubated and placed on mechanical ventilation with concern for septic shock. The patient was immediately started on a norepinephrine drip for blood pressure support, with successful weaning of vasopressors within 48 hours of his ICU stay. Blood and sputum cultures returned positive for vancomycin-resistant, methicillin-sensitive *Staphylococcus aureus*, and he was started on a four-week regimen of IV cefepime based on culture sensitivities. Because of prolonged mechanical ventilation requirements, a tracheostomy was placed.

**Figure 1:**
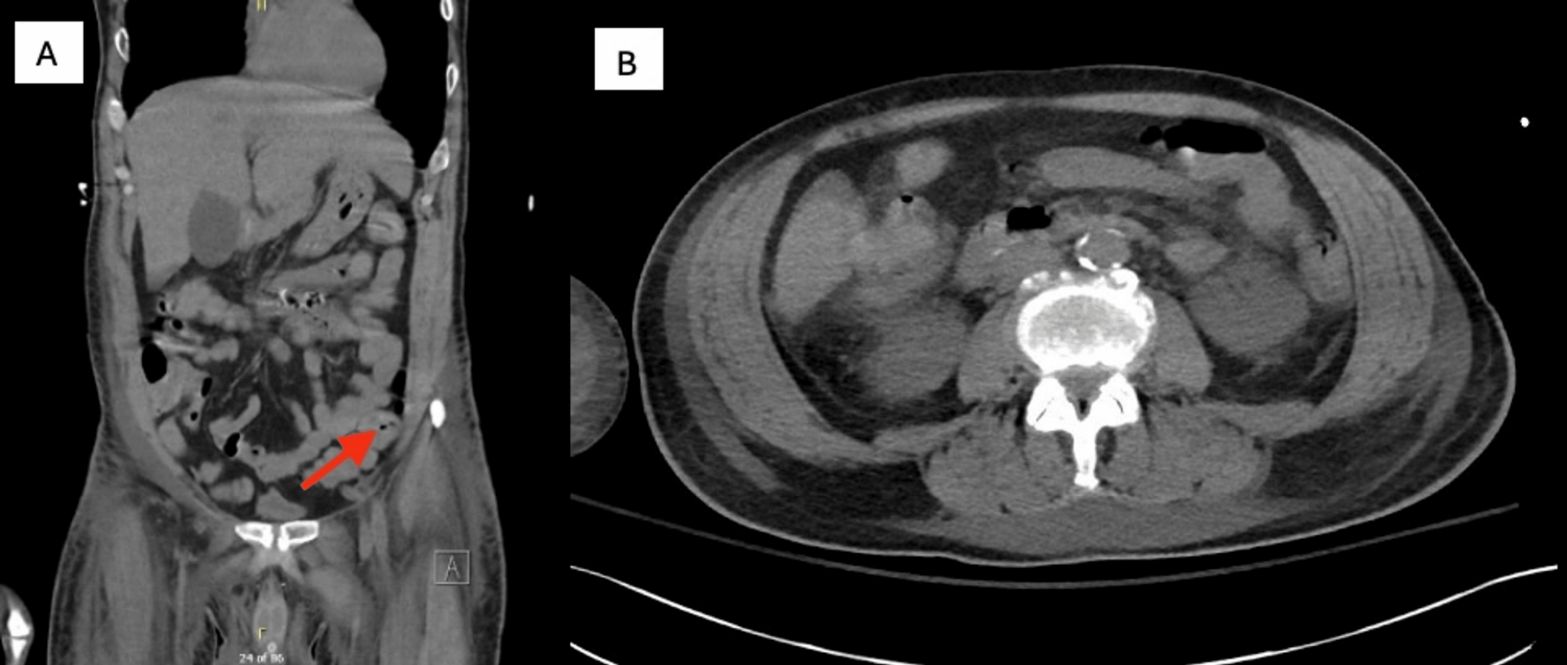
Computed tomography scan of the abdomen and pelvis Computed tomography scan of the abdomen and pelvis, completed on 2/6/2024, includes coronal (A) and axial (B) views. A colonic diverticulum is noted, as shown by the red arrow in (A); the duodenal diverticulum is not pictured in these specific slices of the scan.

On day 6 of the patient’s ICU stay, the patient was noted to have diarrhea with positive *C. difficile* by polymerase chain reaction (PCR), but negative for enzyme immunoassay (EIA) toxin. Despite oral vancomycin treatment, he continued to have fever and significant leukocytosis. A chest and abdominal computed tomography (CT) scan was performed. Chest CT was notable for worsening necrotizing pneumonia of the right upper lobe and a new right-sided pleural effusion with loculations. Abdominal CT did not demonstrate any identifiable infection in the abdomen or pelvis but was notable for colonic diverticulosis and duodenal diverticulum (Figure 1). A peripherally inserted central catheter (PICC) line was inserted, and IV metronidazole was added to the patient’s regimen because of fever, despite a few days of oral vancomycin coverage for *C. difficile* colitis. After several days of this regimen, the patient’s fecal management system (FMS) showed improvement in his diarrhea, and the FMS was removed with minimal further diarrhea.

**Figure 2:**
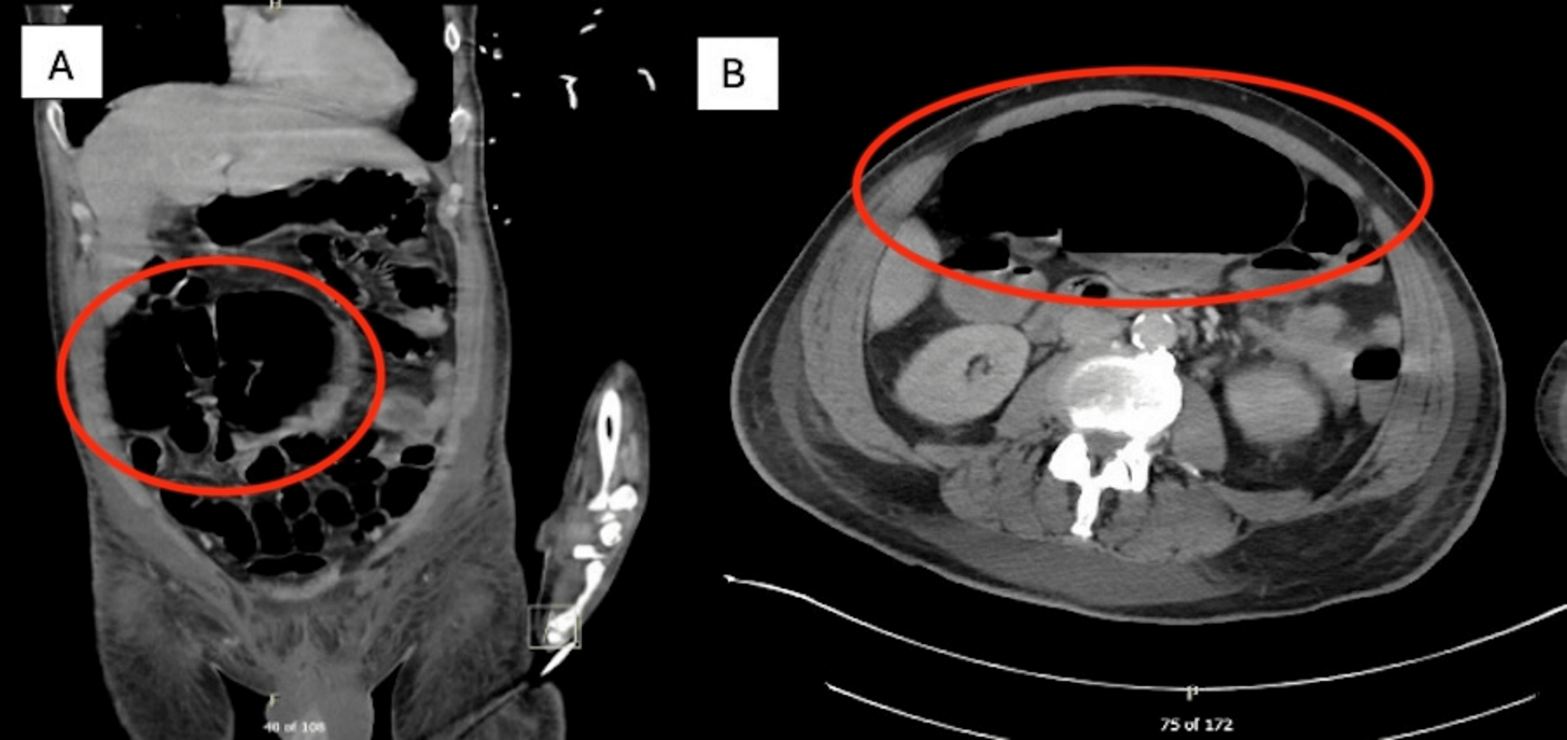
Computed tomography scan of the abdomen and pelvis Computed tomography scan of the abdomen and pelvis, completed on 2/11/2024, includes coronal (A) and axial (B) views. Gaseous distension of the cecum is seen in (A) and (B), as depicted by a red circle.

One day after the removal of the FMS, the patient complained of abdominal discomfort despite tolerating tube feeds via a nasogastric tube. On physical examination, the patient exhibited new-onset abdominal distension, tympany, and tenderness to palpation in the right lower quadrant of the abdomen. The follow-up abdominal CT scan was notable for new-onset gaseous distension of the cecum without focal luminal narrowing (Figure 2). Because of this finding, the patient was placed on bowel rest, and daily supine abdominal X-rays to monitor the status of colonic dilation were ordered. Despite two days of bowel rest and nothing by mouth (NPO) status, the abdominal X-ray was notable for worsening gaseous distension of the cecum, with normal caliber of the remaining colon, favoring an ileus. Persistent and increasing gaseous distension of the cecum despite continued conservative management led to general surgery consultation early in the course of the patient’s hospitalization. At this time, examination of the patient was notable for increased abdominal distension exhibiting any peritoneal signs, such as rebound tenderness or guarding. General surgery recommended urgent surgical intervention with exploratory laparotomy and right hemicolectomy with ileostomy to prevent ischemia and perforation of the cecum.

Upon entering the peritoneal cavity, a cecal bascule was noted immediately, with the surgeon describing the cecum as extremely dilated, with a small serosal tear but without frank perforation. The cecal bascule was released, and the right colon was removed with an ileocolic anastomosis and a diverting loop ileostomy. An end-ileostomy was not performed because the surgeon felt that reversing the loop ileostomy would be less traumatic and technically less problematic than performing an end-ileostomy, which would require a laparotomy with a side-to-side anastomosis versus a simple loop closure. The pathological report of the specimen was notable for dilation of the terminal ileum, marked dilation of the cecum, mild dilation of the ascending colon, and the remainder of the specimen being normal in caliber. The cecal mucosa was noted to be diffusely flat with focal hyperemia, no polyps or mass, and a grossly unremarkable appendix. The cecal wall was noted to be remarkably thin, with diverticulosis. Three enlarged pink lymph nodes were resected, which were otherwise unremarkable on histological examination.

The patient tolerated postoperative care well, with successful draining and normal functioning of the loop ileostomy. On postoperative day 3, the patient developed an acute right upper extremity deep vein thrombosis within proximity to his PICC line, which was confirmed by ultrasound. The patient also developed a deep vein thrombosis in the right posterior tibial vein two days later, confirmed with ultrasound. Therefore, an IV heparin infusion was initiated and subsequently transitioned to oral apixaban.

He continued to improve from postoperative recovery and was liberated from mechanical ventilation. He was transferred to the medical floor and subsequently discharged home with Home Health Care. He remained clinically stable, with a functional ileostomy and tracheostomy, and was tolerating his pre-hospital diet upon discharge. The prognosis in this case was not expected to differ significantly from that of other cases of cecal bascule managed surgically.

## Discussion

We report a case of cecal bascule that was heralded by the development of abdominal distension following the resolution of *C. difficile* colitis. The goal of this case presentation was to describe a complicated case of a cecal bascule that developed throughout hospitalization following suspected infection by *C. difficile* colitis. The cecal bascule is the most infrequent subtype of cecal volvulus and is described as the anterosuperior displacement of the cecum onto the ascending colon, leading to intestinal obstruction and cecal dilation [1]. There is literature describing an association between large bowel obstruction and colonic pathologies such as diverticulitis [4], inflammatory bowel disease [5], *M. tuberculosis* [6], and cytomegalovirus colitis [7]. A case report in 2022 describes the first case of cecal bascule found in a COVID-19-positive patient with leukocytosis and elevated inflammatory markers [9]. However, there is limited literature describing cecal volvulus or cecal bascule in patients with recent colitis, which appears to be the provoking factor for cecal bascule in our patient. A case report from 2019 describes cecal bascule formation secondary to acute cytomegalovirus colitis and diarrhea [7]. That report concluded that cytomegalovirus colitis may affect colonic motility more than just causing diarrhea, leading to pseudo-obstruction and volvulus [7]. Similarly, a case report in 2011 describes the acute development of a cecal bascule after a colonoscopy that visualized segmental colitis [8], further suggesting that inflammation may be a possible etiology of cecal bascules.

Cecal bascule most frequently presents with typical symptoms of bowel obstruction: abdominal distension, abdominal pain, nausea, and vomiting [1]. However, presenting symptoms can range from vague abdominal pain to classic obstructive symptoms [1]. Abdominal pain is the most important presenting symptom of cecal bascule, as repeated cecal obstruction caused by cecal mobility can cause intermittent bouts of abdominal pain that spontaneously resolve [1]. The abdominal pain associated with cecal bascules can be either diffuse or localized to the region of the bascule [2].

An abdominal CT scan is the preferred imaging technique to diagnose a suspected cecal bascule [3]. The typical radiological finding for a cecal volvulus is the “whirl” sign [10]. However, because cecal bascules lack mesenteric torsion, this finding is often absent in CT imaging of cecal bascules [2]. A common finding on a CT scan of cecal bascules is the superomedial displacement of the cecum and ileocecal valve toward the upper abdomen [2]. Because of the difficulty of identifying a cecal bascule on radiological imaging modalities, many cecal bascules are ultimately diagnosed during an exploratory laparotomy [1].

*C. difficile* represents the leading cause of antibiotic-associated diarrhea in the developed world [11]. When *C. difficile* proliferates in the gut, it can produce two exotoxins (toxins A and B), which damage the intestinal epithelium of the gastrointestinal tract, resulting in the characteristic secretory, watery, large-volume diarrhea [12]. The diagnosis of *C. difficile* infection (CDI) is based on several factors, including clinical and laboratory findings [12]. The gold standard for detecting toxigenic *C. difficile* is a positive toxigenic culture in the stool [9]. Current guidelines recommend multistep diagnostic algorithms for CDI, noting that toxin EIA positivity is not mandatory when clinical suspicion for CDI is high [13].

Severe *C. difficile* can notably result in a non-mechanical bowel obstruction, known as toxic megacolon. Severe transmural mucosal inflammation from *C. difficile* colitis triggers this ileus, which can result in colonic dilation of more than 6 cm [14]. Although there is a known association between CDI and toxic megacolon, there is limited evidence of an association between CDI and mechanical bowel obstructions. One case report describes a small-bowel CDI that led to small-bowel obstruction, managed conservatively [15]. To our knowledge, this report is the first case documented of a patient with clinically suspected CDI who ultimately developed cecal bascule during their hospitalization. This case report further raises the question of whether a *C. difficile*-associated bowel obstruction may have contributed to the development of the cecal bascule described in this patient.

Delaying the diagnosis of cecal bascules results in an increased risk of complications, including, but not limited to, ischemia and necrosis of the bowel, cecal perforation, peritonitis, septic shock, and death [1]. Early recognition of presenting symptoms of cecal bascules can aid in earlier diagnosis and management. The authors of this report recommend that clinicians continue to stay vigilant and maintain a widened differential diagnosis when patients present with bowel obstruction symptoms, especially intermittent abdominal pain. We also recommend that clinicians continue to suspect cecal bascule in symptomatic patients despite the absence of classic cecal volvulus radiological findings. Further understanding of cecal bascule presentation, management, and comorbidities has the potential to significantly influence patient outcomes.

## Conclusions

This is a rare, reported case describing the development and findings of a severely dilated cecal bascule in a patient with the recent resolution of suspected CDI. Findings from this case suggest that a *C. difficile*-associated bowel obstruction may play a causative role in the development of cecal bascules. This case highlights the need for heightened clinical awareness of atypical gastrointestinal complications in patients with CDI and contributes to the growing body of evidence suggesting that infection-related alterations in colonic motility may predispose patients to mechanical pathologies such as cecal bascules. Early diagnosis of cecal bascules is crucial because of the risk of severe complications, including ischemia, necrosis, perforation, peritonitis, septic shock, and death, emphasizing the need for clinicians to maintain a broad differential diagnosis and consider cecal bascules, even without typical radiological findings, to improve patient outcomes. 
